# Fungal Community Successions in Rhizosphere Sediment of Seagrasses *Enhalus acoroides* under PAHs Stress

**DOI:** 10.3390/ijms160614039

**Published:** 2015-06-18

**Authors:** Juan Ling, Yanying Zhang, Meilin Wu, Youshao Wang, Junde Dong, Yufeng Jiang, Qingsong Yang, Siquan Zeng

**Affiliations:** 1 Key Laboratory of Tropical Marine Bio-resources and Ecology, South China Sea Institute of Oceanology, Chinese Academy of Sciences, Guangzhou 510301, China; E-Mails: lingjuan@scsio.ac.cn (J.L.); zyy@scsio.ac.cn (Y.Z.); coolj_2007@126.com (Y.J.); s0909501162@163.com (Q.Y.); zengsiquan@126.com (S.Z.); 2 Hainan Tropical Marine Biological Research Station, Chinese Academy of Sciences, Sanya 57200, China; 3 State Key Laboratory of Tropical Oceanography, South China Sea Institute of Oceanology, Chinese Academy of Sciences, Guangzhou 510301, China; E-Mails: mlwu@scsio.ac.cn (M.W.); yswang@scsio.ac.cn (Y.W.)

**Keywords:** seagrass, fungi, PAHs (polycyclic aromatic hydrocarbons), PCR-DGGE (polymerase chain reaction-denaturing gradient gel electrophoresis), qPCR (quantitative PCR), RDA (redundancy analysis)

## Abstract

Seagrass meadows represent one of the highest productive marine ecosystems and are of great ecological and economic values. Recently, they have been confronted with worldwide decline. Fungi play important roles in sustaining the ecosystem health as degraders of polycyclic aromatic hydrocarbons (PAHs), but fewer studies have been conducted in seagrass ecosystems. Hence, we investigated the dynamic variations of the fungal community succession under PAH stress in rhizosphere sediment of seagrasses *Enhalus acoroides* in this study. Polymerase chain reaction-denaturing gradient gel electrophoresis (PCR-DGGE), quantitative PCR (qPCR) and a clone library have been employed to analyze the fungal community’s shifts. Sequencing results of DGGE and the clone library showed that the predominant species belong to phyla Ascomycota and Basidiomycota. The abundance of three groups decreased sharply over the incubation period, whereas they demonstrated different fungal diversity patterns. Both the exposure time and the PAH concentrations affected the microbial diversity as assessed by PCR-DGGE analysis. Redundancy analysis (RDA) indicated that significant factors driving community shifts were ammonium and pH (*p* < 0.05). Significant amounts of the variations (31.1%) were explained by pH and ammonium, illustrating that those two parameters were the most likely ones to influence or be influenced by the fungal communities’ changes. Investigation results also indicated that fungal communities in seagrass meadow were very sensitive to PAH-induced stress and may be used as potential indicators for the PAH contamination.

## 1. Introduction

Seagrasses are the only true flowering plants that grow and reproduce underwater in shallow coastal waters, and they are widely distributed. There are about 72 species of 12 genera of seagrasses throughout the world in a wide variety of habitats [1,2]. Seagrass meadows are characterized by high marine biodiversity and productivity and play a critical role in the equilibrium of coastal ecosystems and human livelihoods [2]. They can stabilize sediments, purify waters and provide food and habitats for marine creatures. It is estimated that 70% to 90% of commercial fish depend on seagrass meadows for a part of their life period [3]. Seagrass plants can also photosynthesize by taking in carbon dioxide and releasing oxygen, so they can potentially help to alleviate rising carbon dioxide levels and contribute to global climate change [4]. They can also exert great importance in the nitrogen and carbon biogeochemical cycles [5,6]. However, seagrass meadows have been at risk over the past few decades.

Previous investigations indicated that the seagrass habitat is declining worldwide. Waycott *et al.* [7] reported that the disappearance rate of seagrass has accelerated from less than one percent per year before 1940 to seven percent per year since 1990, and from the year 1979, 29% of the recorded seagrass areas have disappeared. Seagrass declines may be caused by natural causes, such as grazing, global climate change, sedimentation, erosion and disease and by anthropogenic factors, such as prop scarring, dredging, eutrophication, siltation and toxic chemicals [2]. Among the toxic pollutants, polycyclic aromatic hydrocarbons (PAHs) are thought to be the most ubiquitous pollutants in that they are of great environmental and human health concerns due to their widespread occurrence in marine environment, persistence and carcinogenic properties [3,8]. PAHs have been precisely classified by groups according to the temperature at which they form or their origin, and anthropogenic PAHs can be pyrogenic or petrogenic in origin [8,9]. Pyrogenic PAHs are mainly caused by incomplete combustion of organic matter (e.g., coal, petroleum and wood), while the petrogenic PAHs are derived mostly from crude oil and petroleum products (e.g., kerosene, gasoline, diesel, lubricating oil and asphalt) [8,9]. A comparative survey for chemical quality conducted in Florida seagrass beds suggested that toxic substances accumulated in seagrass-rooted sediments, the concentrations of which were significantly greater than those in adjacent non-vegetated sediments [3]. Furthermore, PAHs tend to be accumulated in sediments and plant tissue, which may lead to adverse results, and are transferred along the food chain [8].

Microbes, including fungi, play an important role in sustaining ecosystem health through functioning both as important contributors and transformers. They can act as plant pathogens and mycorrhizal symbionts. Previous studies showed that fungi could degrade PAHs by co-metabolizing PAHs to a wide variety of oxidized products and, in some cases, to CO_2_. Additionally, both ligninolytic fungi and non-ligninolytic fungi can contribute to the degradation of PAHs [10]. Seagrass *Posidonia oceanica* harbored rich fungal assemblages and plays a central role in the element biogeochemical cycle by decomposing organic matter [11,12].

The report of Apostolopoulou *et al.* (2012) [9] showed that seagrass *Posidonia oceanic* could be used as a recorder of ambient trace metal pollution and a bioindicator of spatiotemporal organic pollution trends. However, scarce information is available regarding the response of fungal communities in seagrass sediments to PAHs [13]. Hence, the present study was therefore undertaken with the aim of: (1) investigating the fungal community in *Enhalus acoroides* sediment by constructing a clone library, and the clone library sequencing results reveal the predominant fungal species and phylogenetic community structure; (2) determining the effects of a mixture of three PAHs, naphthalene (Nap), fluorene (Flu) and pyrene (Pyr), on the fungal community of seagrass sediments through polymerase chain reaction-denaturing gradient gel electrophoresis (PCR-DGGE) and quantitative PCR assay (qPCR) through a 28-day laboratory incubation experiment and distinguishing the effects of the different concentrations of PAHs on fungal communities, as well as the temporal shift of fungal communities as determined from the DGGE bands and fungal 18S rRNA genes copies; (3) analyzing which parameters would be the critical one(s) affecting the fungal distribution during the incubation period.

## 2. Results and Discussion

### 2.1. Sediment Characteristics

Total phosphorus (TP), total nitrogen (TN) and total carbon (TC) contents and the C/N ratio for the sediment sample are summarized in Table 1. TP, TC and TN contents were 0.0035, 0.021 and 0.0384 by dry weight, respectively. Previous investigations suggested that total organic carbon is a very important variable related to the adsorption and persistence of PAHs in marine sediments [14]. The typical C/N values reported for most coastal sediments (C/N = 6 to 10), with the C/N ratios being much higher than the typical C/N values in Xincun Bay sediments, indicated that many inputs of organic carbon were received and that this was highly influenced by terrestrial and anthropogenic sources [15]. Northcott and Kevin [16] reported that organic pollutants in sediments would be controlled by organic matter when the TC content was above 0.1 percent.

**Table 1:** The original organic matter content of the sediment. TP, total phosphorus; TN, total nitrogen; TC, total carbon.

Sample	TP (%)	TN (%)	TC (%)	C/N Ratio
*In situ* Sediment	0.0035	0.021	0.384	18.29

### 2.2. DGGE (Denaturing Gradient Gel Electrophoresis) Patterns the Fungal Communities under PAH (Polycyclic Aromatic Hydrocarbon) Stress

**Figure 1:**
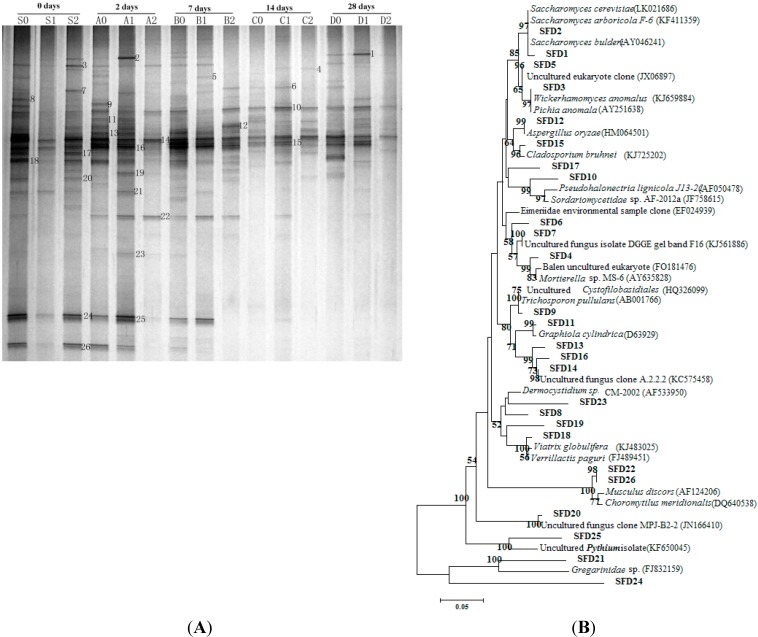
DGGE (denaturing gradient gel electrophoresis) profiles of sediment fungal communities exposed to different concentrations of PAH (polycyclic aromatic hydrocarbon) contamination at different incubation stages (S: Day 0; A: Day 2, B: Day 7, C: Day 14, D: Day 28; 0: control without PAH addition; 1: 100 mg/kg; 2: 1000 mg/kg) (**A**); neighbor-joining phylogenetic tree based on 18S rRNA gene sequences from DGGE bands. Bootstrap analysis was based on 1000 replicates. Bootstrap values from distance analysis are depicted. Bootstrap values less than 50% are not shown (**B**).

The investigation conducted by Sakayaroj *et al.* [17] confirmed that the fungal assemblages of seagrass *E. acoroides* were very rich. The three experimental groups, each in triplicate, were carried out by spiking a mixture of Nap, Flu and Pyr into the fresh seagrass sediment slurries from Day 0 (the beginning) to Day 28 (the end of the experiment). Group Control Check (CK) received no PAH addition, while the PAH concentrations of Group 1 and Group 2 were 100 and 1000 mg·kg^−1^, respectively. Samples were harvested on five different dates, namely S (Day 0), A (Day 2), B (Day 7), C (Day 14) and D (Day 28). In this study, the DGGE profile of 15 samples collected at five different incubation phases are shown in Figure 1A. Differences in the compositions of fungal communities were observed in the same group with the different incubation periods, with some bands obtained at a certain stage, whereas some bands were present through the whole incubation time. For instance, Band 14 could be detected through the 28-day incubation time, while Bands 25 and 26 only can be found at the early stages. By comparison, some bands, such as Bands 2, 4, 5, 19 and 21, could only be detected in Group 1 and/or 2, which indicated that such species can adapt to the PAH-contaminated environment. They may have high endurance with respect PAHs. Previous studies have proven that fungi would be necessary to efficiently remediate multiple polluted sites, and fungi obtained from mangrove sediments have high pyrene-degrading activity, even at an acidic pH of approximately 4 [10,18]. The result is consistent with the report of Wu *et al.* [19] that fungi could degrade the PAHs and use them as carbon sources for growth.

The DGGE bands were labeled “Seagrass Fungi DGGE (SFD)” in the phylogenic tree construction (Figure 1B). Twenty-six bands were excised from the DGGE gel and sequenced. Taxonomic analysis based on top BLAST hits in GenBank showed that all of the DGGE bands sequenced were identified as related to phyla Ascomycota and Basidiomycota and unidentified species (Figure 1B). For instance, Band 2 was more intensive in Group 1 than Group CK, and this may illustrate that the PAH addition stimulated its growth. Sequencing results showed the closest relative of Band 2 in the NCBI database is *Saccharomyces cerevisiae* (LK021686). This fungus also showed high similarity with *Saccharomyces cerevisiae* F-6 (99%) (Figure 1B), and Sutherland [20] and Deng *et al.* [21] have already proven that *S. cerevisiae* had the ability to oxidize PAHs. Likewise, species SFD2 detected in this study might also own the ability to degrade PAHs. Many of the band sequence results were related to uncultured fungi, and they suggest that seagrass sediment harbored a surprisingly rich fungal diversity. Salvo *et al.* [22] have found that there exists a correlation between the abundance of cultivable fungi and the concentrations of PAHs in harbor sediments. Fungi have already been applied to degrade hazardous organic waste, such as *Aspergillus oryza*, which has been reported to accumulate heavy metal ions in its mycelial mass [23]. The BLAST results showed that the species SFD12 had 100% similarity with *A. oryza.* In addition, species SFD12 was intense in Lane B2 after seven days of incubation, which indicated that this fungus could survive and grow under stress induced by high levels of PAHs.

Investigations on the fungal diversity of seagrass meadow recently have been mainly conducted by a culture-dependent method and focus on the fungal endophytes [24,25,26]. The results of Panno *et al.* [25] demonstrated that the fungal assemblage had habitat specificity, with only two species (*Penicillium chrysogenum* and *P. janczewskii*) distributed in four parts: leaves, rhizomes, roots and matte. Many fungal isolates derived from seagrass meadow exhibited antimicrobial potential due to their novel bioactive metabolites [26].

All of the sequences obtained in DGGE have been submitted to the GenBank database and are available under the following accession numbers: KP998709 to KP998734.

### 2.3. Dynamics of Shannon Index, Fungal Abundance Analysis

**Figure 2:**
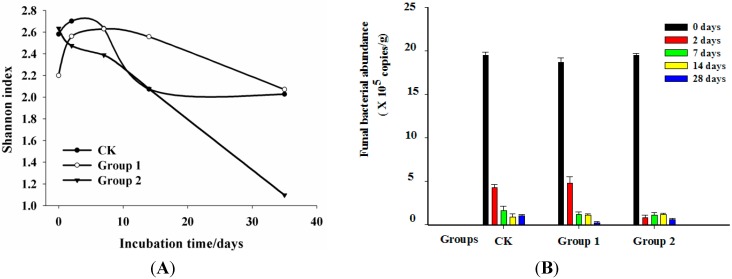
Shannon index for the fungal communities based on the DGGE profile (**A**) and the dynamics of fungal abundance 18S rDNA gene copy number during the incubation (**B**). (S: Day 0; A: Day 2; B: Day 7; C: Day 14; D: Day 28; 0: control without PAH addition; 1: 100 mg·kg^−1^; 2: 1000 mg·kg^−1^).

The patterns of the Shannon index and the abundance of the fungal communities over the whole incubation time presented different tendencies. The Shannon index determined by the DGGE band numbers of all groups changed rapidly though the 28-day incubation period (Figure 2A). In Group CK, it firstly increased from 2.58 to 2.70 in the first two days incubation and then decreased to 2.0 in the end. Group 1 showed a similar tendency of the Shannon index with the CK group in the first 14 days of incubation that firstly slowly increased from Day 0 to Day 2 and then decreased until Day 14. However, the Shannon index of Group 2 dropped sharply from 2.63 to 1.09 through the whole incubation time. The biggest difference among the three groups was that the Shannon index of Group CK increased slowly from Day 14 to Day 28, while Group 1 and Group 2 decreased from the beginning to the end of the incubation. This phenomenon could be explained by the fungi’s capability of utilizing or enduring PAHs to survive in environments with the addition of PAHs, while the others were suppressed or inhibited. This could lead to the reduction of the Shannon index. The results of this study agreed with the microbe of mangrove sediments under contamination by PAH [27].

The abundance of fungal communities in this study as indicated by the number of 18S rDNA copies measured using qPCR is shown in Figure 2B. It is obvious that the abundance of all fungi communities, including Group CK, Group 1 and Group 2, dropped sharply during the incubation period, with the abundance from 1.95 × 10^6^ copies·g^−1^, 1.86 × 10^6^ copies·g^−1^ and 1.95 × 10^6^ copies·g^−1^ to 1.57 × 10^5^ copies·g^−1^, 1.22 × 10^5^ copies·g^−1^ and 1.68 × 10^5^ copies·g^−1^, respectively (Figure 2B). Compared with Group CK, the effect of PAHs in Group 1 was not significant at the earlier stage from Day 0 to Day 2. The promoting effect of low concentrations of PAHs (Group 1) on the fungal growth could be detected in two days of incubation. By comparison, the fungal abundance in Group 2 was higher than in Group 1 at the end of incubation, which indicated that some PAH degraders in the group relied on PAHs when other nutrients were absent. However, the abundance of Group 2 decreased sharply after two days’ incubation. This may due to most of the fungal species being not able to endure such a high concentration of PAHs in short period. Yet, as incubation went on, fungi capable of degrading PAHs could use PAHs as a carbon source in that the abundance of Group 2 increased slowly after Day 2 until Day 14. It could be inferred from this study that fungi were quite sensitive to the PAH addition during the incubation period, especially for the high concentrations of PAHs. Low concentrations of PAHs exerted a promoting effect on fungal growth in the first two days of incubation, while the high concentration of PAHs inhibited the fungal growth in first 14 days and stimulated it from Day 14 to Day 28. Johnsen and Karlson [28] found that a higher content of pollutants might induce and enrich markedly higher species diversity than the groups with a lower content. On the contrary, analysis conducted in mangrove sediments inferred that the low PAH-contaminated group had significantly higher species diversity [27]. Consequently, two different effects could be caused by organic pollutants on microbial diversity in that they could act as carbon sources for microorganisms by stimulating microbial growth or exert their toxic effects on microorganisms and, as a consequence, reduce the species abundance [27,28].

### 2.4. Clone Library Analysis

**Figure 3:**
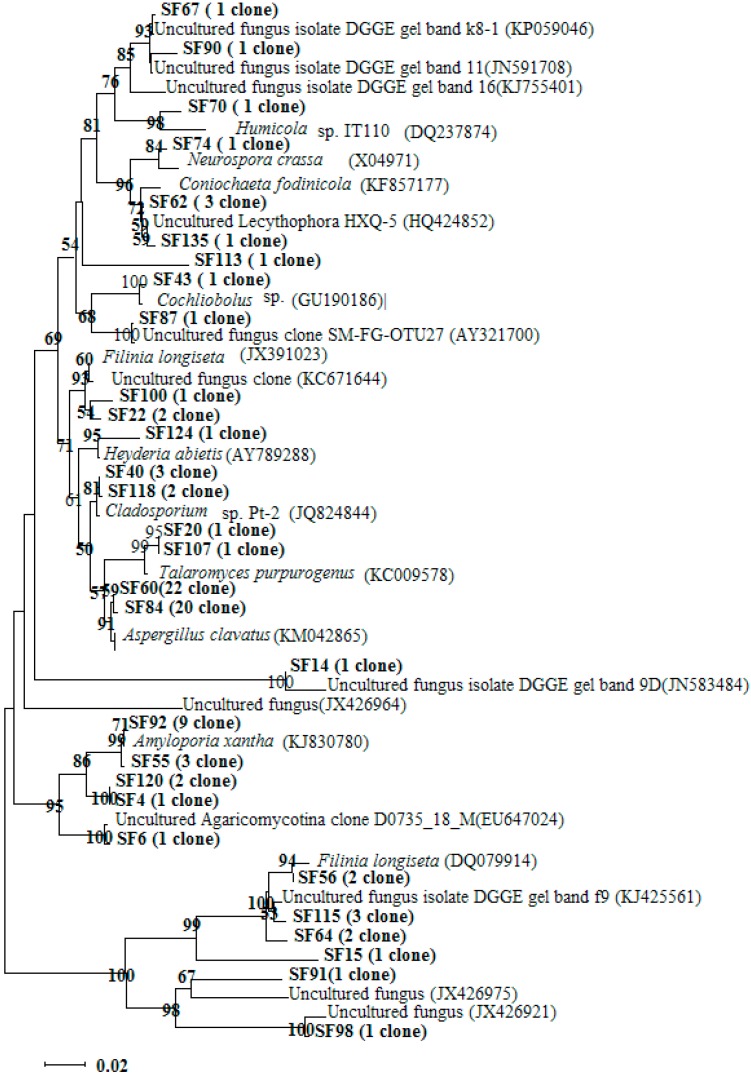
Neighbor-joining phylogenetic tree based on 18S rRNA gene sequences based on the representative clone of each Operational Taxonomic Units (OTU) (cutoff = 0.03) of Seagrass Fungi (SF) clone library Numbers in parentheses indicate clones in each OUT. Bootstrap analysis was based on 1000 replicates. Bootstrap values from distance analysis are depicted. Bootstrap values less than 50% are not shown.

Clone library Seagrass Fungi (SF) has been constructed to reveal the fungal community composition of the native seagrass sediment. One hundred and five clones were randomly picked, and the sequencing results showed that the recombination rate was 96%. All of the sequences obtained in this work have been submitted to the GenBank database and are available under the following accession numbers: KP998609 to KP998708. All of the clones were checked with the online chimera checker program at the Ribosomal Database Project II website (http://rdp.cme.msu.edu). Ninety-six clones were included for further analysis. The Mothur analysis results showed that 43 OTU_0.01_ (operational taxonomic unit (OTU)), 36 OTU_0.02_ and 32 OTU_0.03_, and the coverage was 65%, 76% and 81%, respectively. One representative clone of each OTU_0.03_ was chosen to construct the phylogenic tree with the clone’s numbers in parentheses (Figure 3). Most of the obtained clones were also identified related to phyla Ascomycota (70 clones) and Basidiomycota (16 clones) and unidentified species (10 clones). Fungi belonging to phylum Ascomycota accounted for 73% of all obtained clones, which indicated that they were the predominant fungi in seagrass meadow. Twenty two clones belonging to one OTU had the highest similarity with *Aspergillus clavatus* (KM042865). Many species belonging to genus *Aspergillus* own the ability to degrade PAH, such as *A. fumigatus*, which can cometabolite anthracene [18]. This result also agrees with the report of Sakayaroj *et al.* [17] that fungal groups of the tropical seagrass *E. acoroides* in Thailand were composed of Ascomycota (98%) and Basidiomycota (2%), and from an investigation in Malaysian mangroves ecosystem, of the one hundred and thirty-nine marine fungi identified, 115 were Ascomycota, two Basidiomycota and 22 anamorphic taxa [29]. Consequently phyla Ascomycota and Basidiomycota were prevalently distributed in the marine ecosystem. Covino *et al.* [30] found that all fungal isolates from an oil-polluted soil were Ascomycetes, and some species displayed the ability to use PAH as the sole carbon source for survival. Moreover, Annette *et al.* [31] and Totsche *et al.* [32] reported that *Pleurotus ostreatus* belonging to phylum Basidiomycota has a high efficiency in degrading high molecular weight (HMW) PAHs by producing extracellular enzymes. Many fungi derived from marine environments within phylum Basidiomycota have demonstrated their ability to degrade and detoxify lignocelluloses [33]. The removal efficiencies of yeast for low molecular weight (LMW) and HMW compounds were 89.3% to 98.6% and 66.3% to 89.4% within six weeks, respectively, which demonstrated great potential applications in the field of environmental remediation [33]. In addition, as reported by Panno *et al.* [11], fungi are also active in producing laccases, peroxidases and tannases. Fungi associated with seagrass play an important role in producing or inducing secondary metabolites, which are of great pharmaceutical value [32]. Consequently, the rich assemblages of fungal resources in seagrass meadow need to be explored and applied in different biotechnological fields.

### 2.5. Redundancy Analysis, Variation Partitioning and Unweighted Pair Group Method with Arithmetic Mean (UMPGA)

**Figure 4:**
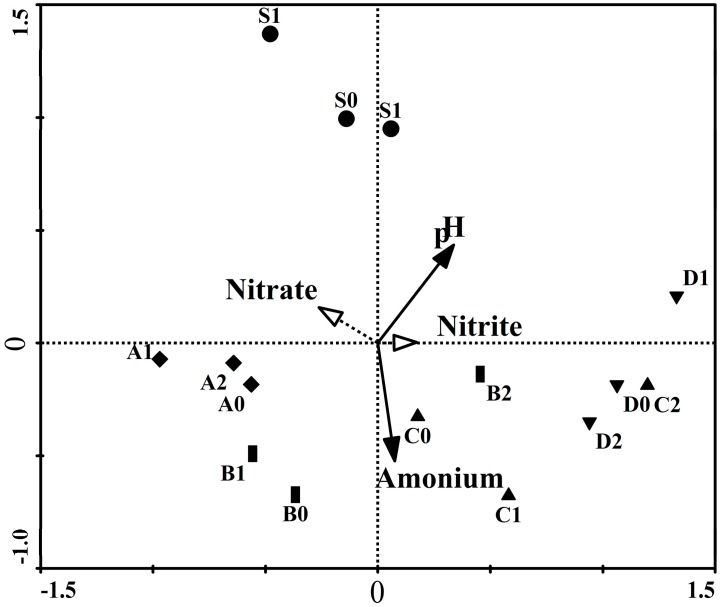
DGGE band data redundancy analysis for fungal communities. Significant composting parameters are indicated by solid lines with filled arrows, while supplementary parameters are shown using gray dotted lines with unfilled arrows. Samples with the same symbol were collected on the same date.

The DGGE fungal community fingerprints were analyzed by redundancy analysis to investigate to what extent the four parameters (pH, nitrate, nitrite and ammonium) caused the variations (Figure 4). These results were all summarized in Table 1. Carlo permutation tests showed that the first and all canonical axes were highly significant (*p* < 0.05), suggesting that these physico-chemical parameters played important roles in driving the shifts of the fungal community composition. The significant factors were pH and ammonium. The first two axes of the RDA ordination indicated the species-environment relationships as 86.4% and 86.5%. The first two canonical axes could explain 27.9% and 8.1% of the variations in the species data, respectively. These results indicated that 45.6% of all variations of the fungal communities during the incubation period were caused by environmental factors (Table 2). Variation partitioning analysis indicated that pH and ammonium posed the predominant effects on the variations of fungal community (Table 3). Fungi play a larger role in N transformations, such as ammonia oxidation, but they are not able to mediate ammonia oxidation in the surrounding environment, so the ammonium concentration in the flasks could influence the growth [34]. pH can affect fungi via controlling the availability of the nutrients that they need, toxicants and the tolerance of organisms, so those species that have the ability to use PAHs could survive when nutritional deficiency occurs in the habitat [35]. In addition, Nielsen *et al.* [36] reported that the low oxygen levels and high salinity could exert an effect on fungal colonization of seagrass. Consequently, more parameters in future investigations should be monitored during the incubation process and included for the further variation of the partitioning analysis.

**Table 2:** Redundancy analysis results of surface and bottom fungal DGGE profiles ^a^.

Axis	Eigen Value	Species-Environment Correlation	Cumulative % Variations of Species	Cumulative % Variations of Species-Environment	Sum of All Canonical Eigenvalue
-	-	-	-	-	0.456
Axis 1	0.279	0.864	27.9	61.3	-
Axis 2	0.081	0.865	36.0	79.0	-
Axis 3	0.062	0.790	42.2	92.6	-
Axis 4	0.034	0.735	45.6	100.0	-

^a^ Monte Carlo significance tests for fungal data: sum of all Eigen values, 1.000; significance of first canonical axis, *F-*value = 3.879, *p* = 0.0020; significance of all canonical axes, *F-*value = 2.097, *p* = 0.004.

**Table 3:** Eigenvalues, *F*-values, and *p*-values obtained from the partial RDAs testing the influence of the significant parameters on the fungal community composition.

Parameters Included in the Model	Eigen Value	Variation Explains Solely (%)	*F*-Value	*p*-Value
Ammonium	0.173	37.90	3.010	0.004
pH	0.207	45.40	3.879	0.002
All the above together	0.311	68.20	2.097	0.004

Partial RDAs based on Monte Carlo permutation (*n* = 499) kept only the significant parameters in the models. For the partial model, the other significant parameters were used as covariables. *F*- and *p*-values were estimated using Monte Carlo permutations. The sum of all Eigen values for both partial RDAs was 1.000.

## 3. Experimental Section

### 3.1. Sample Collection

Sediments of the tropical seagrass *E. acoroides* were collected from Xincun Bay (18°24′34″ N to 18°24′42″ N, 109°57′42″ E to 109°57′58″ E), Hainan Island, China. Xincun Bay with an area of more than 13.1 km^2^ is an enclosed bay with only one narrow channel connected with the open South China Sea. Seagrasses are widely distributed at the south of the lagoon, and *E. acoroides* was one of the predominant species there [37]. Surface sediment samples of seagrass *E. acoroides* (around 0 to 4 cm) were collected in triplicate with a five point method at low tide from the mid-intertidal zone of Xincun Bay on 24 April 2012. All samples were thoroughly homogenized using a sterilized spoon. Then, the sediment samples, both for molecular analysis and sediment characteristics analysis, were transferred to the lab in sterile polyethylene bags in an ice box and then kept frozen until processing.

### 3.2. Experimental Setup

The sediments were processed according to the recommendations of Zhou *et al.* [27] and Wu *et al.* [38] with some modifications, with the first group (Group CK) being the control, while the second (Group 1) and third groups (Group 2) received low and high PAH contamination, respectively. Laboratory-incubated experiments have been designed to minimize the environmental complexity, and the dynamic variations of fungal communities to PAHs were compared. Stock solution of a PAH mixture was prepared by dissolving approximately 1 g of each component (Nap, Flu and Pyr) in 10 mL of acetone. The sediment of the first group was treated with acetone mixed with sterilized water as a control group. In the control (Group CK), 50 g of fresh sieved sediment was added to 100 mL sterilized mineral salt medium (MSM) in a 250-mL Erlenmeyer flask wrapped with aluminum foil with a layer of glass beads. For Group 1, PAHs were added to the flask with a final concentration of 100 mg·kg^−1^ mixed PAHs (33.3 mg·kg^−1^ each for Nap, Flu and Pyr) prior to the addition of sediment and water, while the concentration of Group 2 was 1000 mg·kg^−1^ mixed PAHs (333.3 mg·kg^−1^ each for Nap, Flu and Pyr). The temperature and salinity of all the flasks were adjusted to 30% and 25 °C in accordance with the *in situ* temperature and salinity of the sampling site. The slurry samples (5 mL) were collected from each flask at Days 0, 2, 7, 14 and 28 with a plastic cut-off-tip pipette and then stored at −20 °C prior to being molecularly analyzed. At each sampling point, the pH values were measured using a pH meter (Shanghai, China), and the other nutrients according to the protocols of “The specialties for oceanography survey”. Samples were analyzed for nitrate (NO_3_-N/µmol·L^−1^) with a SKALAR auto-analyzer (Skalar Analytical B.V. SanPlus, Breda, The Netherlands). Ammonium (NH_4_-N/µmol·L^−1^) was analyzed with the method of being oxidized by hypobromite and molybdophosphoric blue. Total phosphorus (TP), total carbon (TC) and total nitrogen (TN) of seagrass sediment were determined by Solid Sample Module SSM-5000A according to standard procedures (ISO 1994) and Zhao *et al.* [14].

### 3.3. DNA Extraction, Clone Library and PCR-DGGE

Total microbial community DNA was extracted using a Soil DNA Kit (Omega, Norcross, GA, USA) according to the manufacturer’s protocol. The DNA purity and concentration were measured using a NanoDrop2000™ spectrophotometer. Primer sets NS1 (5′-GTAGTCATATGCTTGTCTC-3′)/Fung (5′-ATTCCCCGTTACCCGTTG-3′) of the 18S rDNA gene have been employed to amplify the targeted sequences [38]. A clone library was created for analyzing the *in situ* fungal community composition.

Target DNA fragments for clone library construction were amplified in 25-μL PCR mixtures containing the following final concentrations or total amounts: 5 ng genomic DNA, 2 μL 10× PCR buffer, 1.6 μL dNTP (2.5 mM, Takara, Otsu, Japan), 0.8 μL of each primer (10 μM, Invitrogen Trading, Shanghai, China) and 22 μL RNase-free water. Thirty cycles of PCR were then performed by using 95 °C for 30 s, 55 °C for 30 s and 72 °C for 30 s, followed by 72 °C for 10 min. The PCR products were then verified by running a 0.8% (*w*/*v*) agarose gel electrophoresis in 1× Tris-acetate-EDTA (TAE) buffer followed by staining with ethidium bromide, and the resultant PCR product of 337 bp was obtained. Purified PCR products of the obtained bands were purified and then ligated into a pMD18-T cloning vector prior to being transformed into *Escherichia coli* DH5*α* according to the manufacturer’s instructions (Takara Shuzo Co., Ltd., Otsu, Japan). One hundred clones were randomly picked, and then, the colony PCRs were performed with M13 primer on an ABI3730 DNA Sequencer at Shanghai Invitrogen Biotech Co., Ltd. (Shang, China).

DGGE analysis of the amplified genes was performed with primer sets NS1/Fung-GC under the condition described above using the Dcode universal mutation detection system, as described in the manufacturer’s manual (Bio-Rad, Hercules, CA, USA) [39]. Optimal separation of electrophoresis was performed with a denaturing gradient of 45% to 65% (100% denaturant with 7 M urea and 40% formamide) and 6% polyacrylamide gels (ratio of acrylamide to bis-acrylamide, 40:1) submerged in 1× TAE buffer (40 mM Tris, 40 mM acetic acid, 1 mM EDTA, pH 7.5). The DGGE electrophoresis runs were carried out at a constant temperature of 60 °C and maintained at 100 V for 17 h. Dominant band and bands of interest of DGGE were excised from the polyacrylamide gel, re-suspended in 30 μL of Milli-Q water and stored overnight at 4 °C, with a volume of 2 μL of the supernatant used for re-amplification using the original PCR conditions with the same primer pair without GC. The PCR products were purified, then inserted into the PMD18-T vector using recombination techniques and subsequently transformed into *Escherichia coli* DH5α according to the manufacturer’s instructions (Takara Shuzo Co., Ltd., Otsu, Japan). Positive recombinants were then submitted for sequencing with an M13 primer on an ABI3730 DNA Sequencer (USA) at the Shanghai Invitrogen Biotech Co., Ltd.

### 3.4. qPCR

Quantification of dynamic variations of the fungal populations in the five different incubation phases of the three groups was determined by PCR using the same primer set as described above for DGGE, but without GC clamps. Assays were performed in triplicate using with an iQ5 Multicolor Real-Time PCR Detection System using SYBR Green as a fluorescent dye (Takara Bio, Tokyo, Japan). All PCR runs started with an initial enzyme activation step performed at 95 °C for 10 min. Each reaction was performed in a 20-μL volume containing 2 μL of DNA template, 0.1 μL of each primer (10 μM, NS1 and Fung), 10 μL of 2× SYBR^®^ Premix Ex Taq™ (Tli RNaseH Plus) (Takara, Japan), 0.4 μL of ROX Reference Dye (50×) and 7.4 μL of ddH_2_O. The reaction conditions for amplification were 95 °C for 1 min and 40 cycles of 95 °C for 10 s, 55 °C for 30 s and 72 °C for 20 s. A melting curve was then generated using a program of 95 °C for 10 s, 55 °C for 30 s and a subsequent temperature increase to 95 °C. Ten-fold serial dilutions of a known copy number of the plasmid DNA were subjected to the qPCR assay in triplicate to generate an external standard curve. The standard plasmid carrying the target gene fragment was generated by amplifying from extracted total community DNA of sediments and cloning into the pEASY-T Vector (Axygen, Union City, CA, USA). The plasmid DNA concentration was determined a NanoDrop 2000c (NanoDrop Technology, Wilmington, DE, USA), and the copy number of the target fungal gene was calculated directly from the concentration of the extracted plasmid DNA according to cycle threshold (*C*_t_) data [40].

### 3.5. Sequencing, Phylogenetic Analysis and Statistical Analysis

All of the obtained nucleotide sequences were manually proofread, analyzed against sequences in the Ribosomal Database Project (RDP) using the Classier tool and NCBI Taxonomy Browser using the Basic Local Alignment Search Tool (BLAST) program (http://www.ncbi.nlm.nih.gov/blast). The aligned sequences of the 18S rDNA gene of clone library Segrass Fungi (SF) was analyzed with the software Mothur [41] to determine operational taxonomic units (OTUs) at a 1%, 2% and 3% dissimilarity cutoff. Mothur was further used to estimate Chao1, Shannon index, Simpson index and the coverage [41]. The phylogenetic tree of partial 18S rDNA sequences was generated using the neighbor-joining algorithms in Mega IV software with each representative clone of each OUT at a dissimilarity of 3% [42]. The evolutionary distances were computed using the maximum composite likelihood method [43] and expressed as the number of base substitutions per site. The level of support for the phylogenies derived from the neighbor-joining analysis was determined using 1000 bootstrap replicates [44].

The PCR-DGGE banding profile of the fungal communities has been digitized after average background subtraction for the entire gel using the software Quantity One 4.6.2 (Bio-Rad, USA), and the subsequent analyses were performed according to Roesti *et al.* [45] and Zhang *et al.* [46]. CANOCO 4.5 computer software was employed to assess the fungal community composition and physicochemical parameters, and detrended correspondence analysis (DCA) was firstly carried out to decide between a linear or unimodal response model for these microbial data. The length of the first DCA ordination axis was 3.914, so we chose the redundancy analysis (RDA) to ordinate the spatial and temporal compositions of the fungal community in five different incubation phases, and the variation partitioning was also conducted to discriminate the influence of each significant parameter using partial RDA [47]. The statistical significance of the first canonical axis and all of the canonical axes was determined by Monte Carlo reduced model tests using 499 permutations. Statistical significance was kept at *p* < 0.05 for all analyses. Similarities of the fungal community compositions were analyzed through the unweighted pair group method with arithmetic means (UPGMA) based on the Bray–Curtis similarity. The Shannon index of different communities was analyzed according to Zhang *et al.* [48] and Ling *et al.* [49].

## 4. Conclusions

The objectives of this research were to reveal the fungal community compositions and their variations under PAH stress in seagrass *E. acoroides* meadow. A total of 26 bands were excised from the DGGE gel and sequenced, and one hundred and five clones were randomly picked and sequenced. All of the results showed that fungal species from phyla Ascomycota and Basidiomycota predominated in rhizosphere sediment. Previous studies have shown that they have the potential to degrade PAHs. The abundance of fungi communities of Group CK, Group 1 and Group 2 dropped significantly over the whole incubation period, with the abundance from 1.95 × 10^6^ copies·g^−1^, 1.86 × 10^6^ copies·g^−1^ and 1.95 × 10^6^ copies·g^−1^ to 1.57 × 10^5^ copies·g^−1^, 1.22 × 10^5^ copies·g^−1^ and 1.68 × 10^5^ copies·g^−1^, respectively. Group 1 showed a similar Shannon index tendency as the CK group that firstly increased (from Day 0 to Day 2) and then fell at the later incubation stages, while the Shannon index of Group 2 dropped sharply from 2.63 to 1.09 through the whole incubation time. Our findings suggest that a low concentration (100 mg·kg^−1^) of PAH pollution could increase the fungal diversity in a short time, while the high concentration of PAHs (1000 mg·kg^−1^) decreases fungal diversity in a short time and could be utilized for a long period (from Day 7 to Day 28) in the rhizosphere sediment of *E. acoroides*. All of the results obtained from this investigation indicated that fungi were more sensitive to high concentrations of PAHs (1000 mg·kg^−1^). RDA analysis showed that ammonium and pH (*p* < 0.05) were the significant factors for driving the fungal communities’ changes, and they could explain 31.3% of the variations.
